# Age-related changes in arthritis susceptibility and severity in a murine model of rheumatoid arthritis

**DOI:** 10.1186/1742-4933-6-8

**Published:** 2009-06-11

**Authors:** Oktavia Tarjanyi, Ferenc Boldizsar, Peter Nemeth, Katalin Mikecz, Tibor T Glant

**Affiliations:** 1 Section of Molecular Medicine, Departments of Orthopedic Surgery, Biochemistry and Internal Medicine (Rheumatology), Rush University Medical Center, Chicago, Illinois 60612, USA; 2 Department of Immunology and Biotechnology, Faculty of Medicine, University of Pecs, 7643, Hungary

## Abstract

**Background:**

Rheumatoid arthritis (RA) most often begins in females in the fourth-fifth decade of their life, suggesting that the aging of the immune system (immunosenescence) has a major role in this disease. Therefore, in the present study, we sought to investigate the effect of age on arthritis susceptibility in BALB/c mice using the proteoglycan (PG)-induced arthritis (PGIA) model of RA.

**Results:**

We have found that young, 1-month-old female BALB/c mice are resistant to the induction of PGIA, but with aging they become susceptible. PG-induced T cell responses decline with age, whereas there is a shift toward Th1 cytokines. An age-dependent decrease in T cell number is associated with an increased ratio of the memory phenotype, and lower CD28 expression. Antigen-presenting cells shifted from macrophages and myeloid dendritic cells in young mice toward B cells in older mice. The regulatory/activated T cell ratio decreases in older mice after PG injections indicating impaired regulation of the immune response.

**Conclusion:**

We conclude that immunosenescence could alter arthritis susceptibility in a very complex manner including both adaptive and innate immunities, and it cannot be determined by a single trait. Cumulative alterations in immunoregulatory functions closely resemble human disease, which makes this systemic autoimmune arthritis model of RA even more valuable.

## Background

Immunosenescence, the age-related decline of immune function, is associated with a broad spectrum of changes affecting both innate and adaptive immunity [1,2]. The frequency of infections, malignancies, and autoimmune diseases increases with age due to the decline of normal immune surveillance and dysregulation of immune responses [2,3]. Aging is a definitive risk factor for rheumatoid arthritis (RA), and numerous studies focused on the "premature immunosenescence" in RA [3-6]. Although several markers of immunosenescence have been described both in mice and humans, there are still gaps in our understanding; in turn, the incomplete understanding of how immunosenescence gives rise to autoimmunity.

Alteration of the T cell repertoire is a major contributor to the age-related decline of adaptive immunity [3,6,7]. For example, thymus atrophy leads to decreased T cell output [8] and reduced T cell receptor (TCR) rearrangement [4]. This decreased thymic function poses a stress on peripheral T cell expansion [9] simultaneously leading to the rapid shortening of their telomere regions [4,10]. In RA, T cells show evidence of senescence 20–30 years earlier than T cells from healthy individuals [4,5].

On the molecular level, age-related alterations in TCR signaling [11] and the loss of CD28, a key costimulatory molecule on T cells, have been described. CD28null CD4+ T cells are less responsive to regulatory T cell (Treg)-mediated suppression [9,12] and senescent human T cells aberrantly express costimulatory molecules KIR2DS2, NKG2D, and CX(3)CR1, instead of their normal costimulatory molecule CD28 [13,14]. In addition, diminished expression of CD40L, another costimulatory molecule, by aged T cells may not provide sufficient help to B cells [15]. In contrast, increased expression of CD70, has been detected in RA patients, with the potential of lowering the threshold of TCR signaling [16]. Collectively, these abnormalities in T cell activation could facilitate the development of autoimmunity [9,17]. B cells are also affected, and in the process of immunosenescence these cells exhibit a decreased capacity of antibody production against foreign antigens and increased autoantibody production [18]. Impaired humoral response is possibly due to decreased bone marrow output of naive conventional (follicular) B cells, which is compensated in the periphery with marginal-zone-, CD5+ B1-like-, and memory, B cells [18]. Instead of high-affinity IgG type antibodies, the production of low-affinity cross-reactive and often autoreactive IgM antibodies was observed in aging individuals [19]).

Proteoglycan (PG) aggrecan-induced arthritis (PGIA) is a murine model of RA. Repeated intraperitoneal immunizations with human cartilage PG leads to joint inflammation, progressive cartilage destruction, bone erosion and ankylosis in peripheral joints of genetically susceptible mice [20]. The mechanism of the disease is based upon the cross reactive immunity, at both T cell response and antibody production, between the mouse (self) and human PG used for immunization [21-23]. Autoimmune features of PGIA such as autoantibody production as well as the cytokine profile in serum closely resemble RA. The clinical appearance of PGIA, the greater susceptibility of aging female mice, and the importance of genetic predisposition are additional characteristics shared with RA (reviewed in [24]).

Almost two decades ago, we found that aging female BALB/c mice are more susceptible to PGIA than young animals, and since then we have routinely used "retired breeder" females to induce the disease [24,25]. However, the age-related increase in susceptibility to PGIA has never been investigated in a systematical study. Therefore, the aim of this study was to examine the effect of age on PGIA susceptibility in BALB/c mice, and to find immunologic parameters that may contribute to the age-related increase in disease development. Using a standard immunization protocol, here we show, for the first time, that young (1-month-old) BALB/c mice are fully resistant to PGIA; 2–3-month-old mice develop relatively mild arthritis with delayed onset, whereas mice at 4-months of age or older at the beginning of immunizations are highly susceptible to PGIA. Immunosenescence in BALB/c mice is associated with changes in their T cell repertoire. An age-related expansion of CD4+CD28^low ^T cells, together with a shift toward a decreased ratio of regulatory/activated (Treg/Tact) CD4+ T cells in secondary lymphoid organs of PG-immunized aging mice have been found in correlation with a shift in the cytokine balance toward the Th1 phenotype. These complex age-related changes may result in partial loss of control over T cell responses, which, in turn, can lead to autoimmunity and arthritis development.

## Results

### Susceptibility to PGIA increases with age

**Figure 1:**
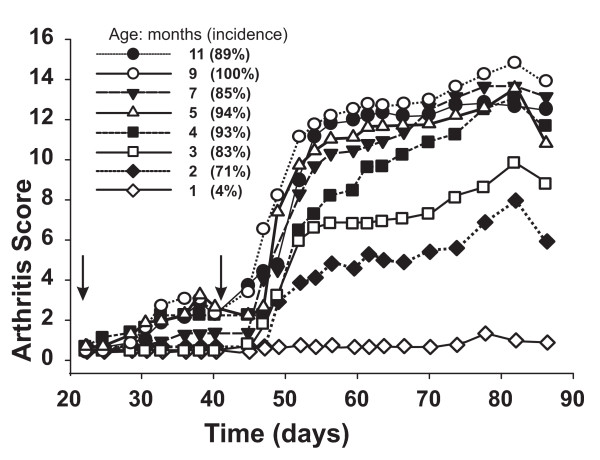
**Development of proteoglycan (PG) induced arthritis (PGIA) in female BALB/c mice immunized with PG at different ages**. Arthritis scores are indicated on the *y*-axis and the experimental period on the *x*-axis. Incidence is shown as percent in brackets next to the symbols of each age group. The first PG immunization was given on day 0, and arrows on the *x*-axis show the second and third PG injections, given on days 21 and 42. Animals were scored 3 times a week, and a visual score from 0 to 4 of each paw was given as described in the Methods. Number of animals in each group sacrificed on day 87 is shown in Table 1.

RA in humans most often begins in the forth-fifth decade of life with female dominance; similar to this, older female BALB/c mice have been found to be more susceptible to PGIA than young females [24,25]. Therefore, we performed a systematic study, using side-by-side immunizations with PG in DDA adjuvant on eight different age groups of BALB/c mice (Figure 1, Table 1). The 4–11-month-old groups developed the highest disease severity (arthritis score: 10.8–14.6) with 85–100% incidence and early onset (Figure 1). The 2–3-month-old mice showed lower incidence (71–83%) and less severe arthritis (arthritis score: 5.8–9.6) accompanied by delayed onset (Figure 1). The 1-month-old group was essentially resistant to PGIA: the disease incidence was less than 5% and the arthritis score was only 1.6 ± 0.3 (Figure 1).

**Table 1:** Serum antibody levels in aging BALB/c mice immunized with human PG

**Age**		**Heteroantibody (mg/ml)** **(anti-human PG)**		**Autoantibody (μg/ml)** **(anti-mouse PG)**	
**(months)**	**n**	**IgG1**	**IgG2a**	**IgG1**	**IgG2a**
**1**	48	**3.32 ± 0.26**	**0.69 ± 0.12**	**41.6 ± 6.0**	**39.6 ± 7.3**
**2**	34	5.61 ± 0.33^†^	1.40 ± 0.24^†^	111.1 ± 8.2*	86.7 ± 9.6*
**3**	30	7.88 ± 0.57^†^	0.66 ± 0.14	108.5 ± 11.7*	104.5 ± 22.5*
**4**	27	5.72 ± 0.44^†^	1.71 ± 0.29^†^	50.0 ± 7.3	91.3 ± 23.5*
**5**	17	6.68 ± 0.47^†^	1.09 ± 0.23	86.3 ± 8.5*	33.6 ± 13.5
**7**	20	6.43 ± 0.61^†^	0.86 ± 0.13	63.1 ± 7.4	45.8 ± 8.8
**9**	28	5.65 ± 0.52^†^	1.05 ± 0.13	65.7 ± 6.6	68.2 ± 14.3
**11**	9	2.50 ± 0.64	0.50 ± 0.11	46.0 ± 15.9	30.4 ± 8.2

Serum levels of PG-specific hetero- and autoantibodies (against human and mouse

PG, respectively) and number of animals in the individual age groups. Age (months) indicate when the first PG injection was given; animals were sacrificed 87 days later, when serum samples were collected. Significantly (p < 0.05) higher ^†^heteroantibody and *autoantibody concentrations compared to the 1-month-old group (bold face) are indicated.

### Evaluation of the age-related changes in the immune response to PG

**Figure 2:**
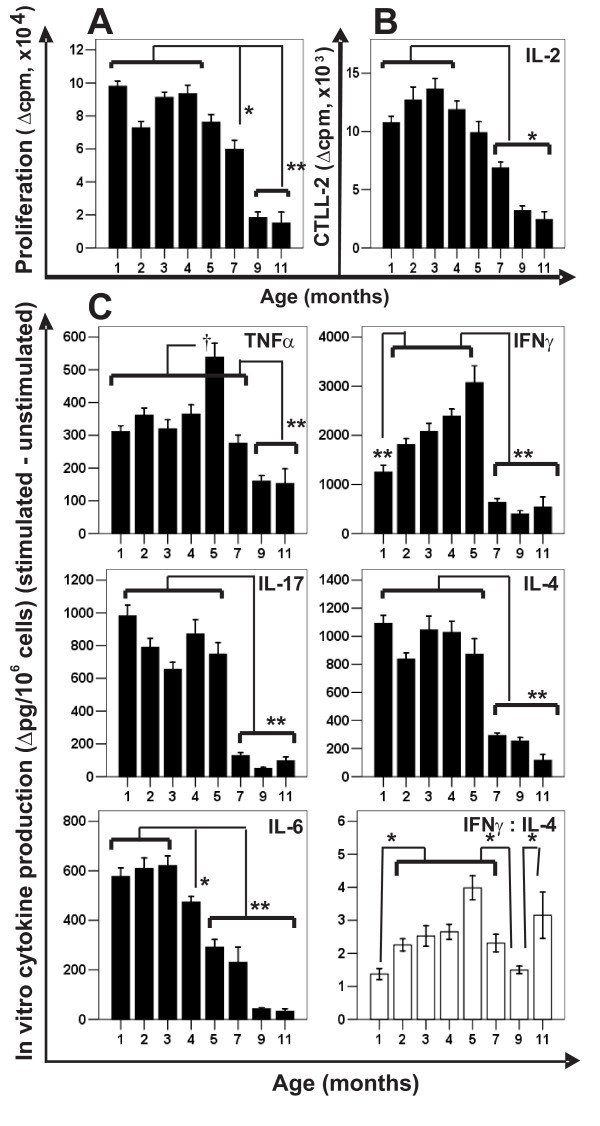
**PG-specific *in vitro *T cell responses in different age groups of PG-immunized mice**. (**A) **PG-specific T cell proliferation (expressed as Δcpm), and (**B**) IL-2 production (expressed as Δcpm of CTLL-2 cells). (**C**)PG-induced *in vitro *production of TNFα, IFNγ, IL-17, IL-4 and IL-6 (Δpg cytokine/million cells). Open bars in the right-bottom corner of Panel C show the IFNγ:IL-4 ratios of the different age groups. Significantly lower (*p < 0.05 or **p < 0.01) or higher (^†^p < 0.05) values are indicated.

To clarify the reason for the profound differences in PGIA susceptibility, first we evaluated the age-related changes in the PG-induced immune responses (*in vitro *T cell response, and serum cytokine and antibody levels). PG-induced *in vitro *T cell proliferation and IL-2 production decreased linearly and significantly with age (Figures 2A and 2B). PG-stimulated spleen cell cultures from older mice (dramatically from the age of 7 months) secreted significantly less IL-4, IL-6, IL-17 and IFNγ (Figure 2C). IL-6 production also showed linear decrease with age; IL-4 and IL-17 levels were similar in the 1–5-month-old groups, and significantly less in older mice (Figure 2C). PG-induced production of IFNγ and TNFα was high in groups between 1 to 5 months of age, and then declined (Figure 2C). The IFNγ: IL-4 ratio, which has been used to characterize the Th1/Th2 balance [26,27], was significantly higher in mice older than 2 months than in the 1-month-old (essentially PGIA-resistant) mice (Figure 2C), although the antigen-specific cytokine production (both IFNγ and IL-4) dramatically declined after 7 months of age.

As potential biomarkers of arthritis in the aging groups, we correlated the serum cytokine and antibody levels with the clinical parameters. Although there were a number of significant differences, none of the measured cytokines in serum showed a clear correlation with the clinical parameters of the corresponding age groups (data not shown). On the contrary, serum antibody levels showed significant positive correlation with disease severity (arthritis score) (Table 1). Notably, the 1-month-old, essentially PGIA-resistant, group had lower antibody concentrations than most of the other groups that had developed arthritis (Table 1).

### Age-related changes in the cellular composition of peritoneal lavage fluid (PLF), spleen, and blood in naive non-immunized mice

To characterize the cell populations that participate in the control of local immunological events, eventually affecting arthritis susceptibility and severity, we analyzed T cells and antigen presenting cells (APCs; B cells, dendritic cells and macrophages) in tissue compartments that were primarily exposed to the antigen. We hypothesized that early activation events should take place in the peritoneum (PLF population), followed by changes in spleen, and blood [28].

**Figure 3:**
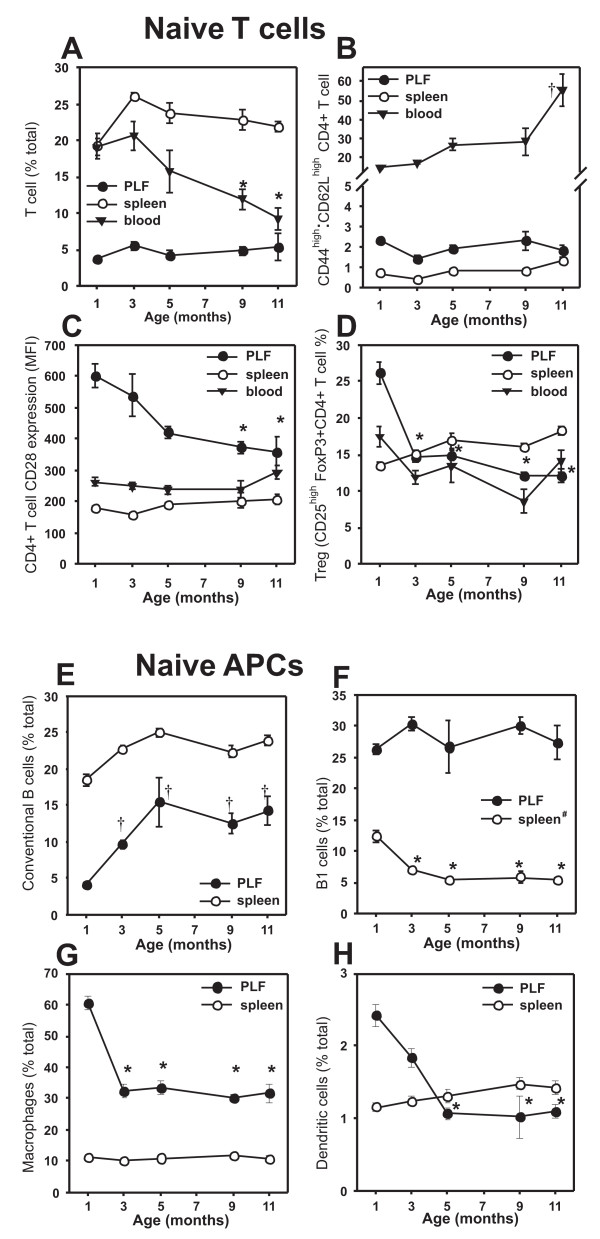
**Age-related changes in the cellular composition of the peritoneal lavage (PLF), spleen, and blood of naive (non-immunized) BALB/c mice**. Upper panels (**A-D**) show the changes of the T cell repertoire: (**A**)T cell percentage, (**B**) CD44^high ^(memory): CD62L^high ^(naive) CD4+ T cell ratio, (**C**) CD28 expression on CD4+ T cells, and (**D**) Treg ratio. Lower panels (**E-H**) summarize the age-related changes in APC composition in the PLF and spleen: (**E**) conventional B cells, (**F**) B1 cells (^#^In the spleen this cell population is comprised of B1 cells, transitional B cells and marginal zone B cells), (**G**) macrophages (F4/80+ cells), and (**H**) dendritic cells (CD11c+ cells). Data represent the mean ± SEM (n = 4–5 mice per group). Values were significantly (p < 0.05) *lower or ^†^higher than those measured in the 1-month-old group as indicated.

In the blood, the percentage of T cells decreased substantially, while the ratio of CD44^high ^(activated/memory):CD62L^high ^(naive) CD4+ T cells increased with age, but both remained at constant levels and ratios in the spleen and the PLF (Figures 3A and 3B). CD28 expression on CD4+ T cells decreased with age in the PLF, but it was similar in all age groups in the blood and spleen (Figure 3C). CD28 expression was highest for PLF CD4+ T cells in 1- to 3-month-old mice (Figure 3C). The percentage of Tregs decreased in the PLF and blood, but increased in the spleen with age (Figure 3D).

In addition to the significant changes in the T cell composition with age, there were alterations in the proportions of APCs as well. The number of conventional B cells increased during the first 5 months of age, especially in the PLF (Figure 3E). B1 cells represented a major population at all ages in the PLF, but their percentage decreased significantly in the spleen between 1 and 3 months of age (Figure 3F). (Note: in the spleen, transitional B cells and marginal zone B cells are also included in the B1 population). The percentage of macrophages and dendritic cells (especially those of myeloid origin, data not shown) decreased between 1 and 5 months of age in the PLF, but remained approximately the same in the spleen (Figure 3G and 3H).

### Regulatory and activated T cell proportions change with age upon PG immunization

There were significant age-dependent changes in the Treg population (Figure 3D). Activation of T cells is a prerequisite for the development of PGIA [29], while Tregs are thought to suppress autoimmunity [30]. Therefore we sought to investigate the effect of PG immunization on the ratio of regulatory (CD25^high^FoxP3+) and activated (CD25^high^) CD4+ T cells (Treg and Tact, respectively) in the different age groups, to determine if the ratio of these cells correlated with the age-related differences in PGIA susceptibility (Figure 1).

**Figure 4:**
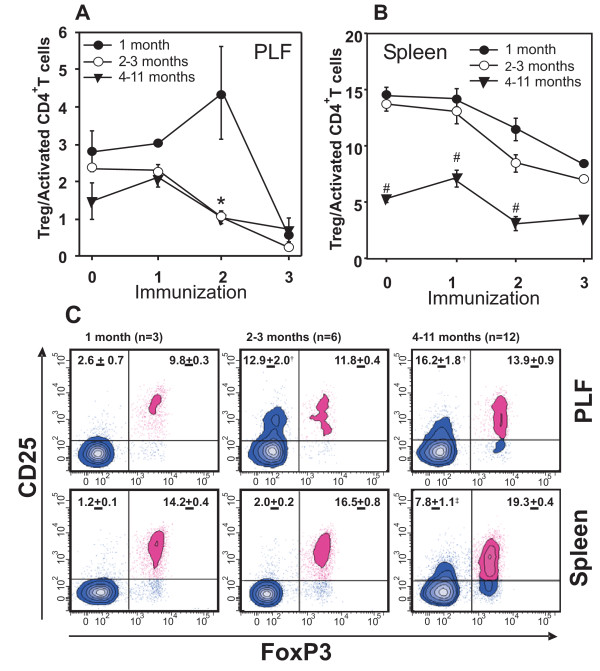
**Changes in the ratio of regulatory (CD25^high^FoxP3^+^) and activated (CD25^high^) CD4+ T cells in (A) the PLF and (B) the spleen of 1-, 2–3-, or 4–11-month-old mice through the immunization period**. Values in the plots show the mean ± SEM calculated from the data of 3–12 mice in each group. Significantly (p < 0.05) lower values in the 4–11-month-old group are indicated [(**A**)*2–3-month-old and 4–11-month-old versus 1-month-old, (**B**)^#^4–11-month-old versus 1-month-old and 2–3-month-old group]. (**C**)Representative flow cytometric contour plots show the distribution of CD4+ T cells based on CD25 and FoxP3 staining 12 days after the second PG immunization in the PLF and spleen. Percent (mean ± SEM) of regulatory (CD25^high^FoxP3^+^) CD4+ T cells (upper right quadrant), and activated (CD25^high^) CD4+ T cells (upper left quadrant) are shown. Values in the plots were calculated from the data of 3–12 mice (as indicated above the panels). Significant (p < 0.05) differences are indicated (^†^2–3-month-old and 4–11-month-old groups versus 1-month-old group; ^‡^4–11-month-old group versus 1-month-old and 2–3-month-old groups).

The Treg/Tact ratio decreased in the PLF by the end of the immunization regime, when compared to the naive mice in all age groups (Figure 4A). However, there was a marked increase in Tregs after the second immunization in the PLF of 1-month-old mice when compared to all other groups (Figure 4A). In the spleen, the Treg/Tact ratio was significantly lower in the 4–11-month-old groups than in the 1-, and 2–3-month-old groups of naive animals, and consistently declined throughout the immunization period (Figure 4B).

Figure 4C shows representative flow cytometry panels and summarizes the regulatory and activated T cell ratios in the PLF and spleen of 1 month-old, 2–3-months-old, and 4–11-months-old mice 12 days after the second i.p. immunization. This time point was chosen because the T cells primed after the first PG/DDA injection, are reactivated after the second injection, and might be involved in the initiation of arthritis [28]. Although the number of Treg cells also increased during aging, the CD25^high^CD4+ (activated) T cell percentage in the PLF and spleen was several-fold higher in 4–11-month-old mice than in the younger mice after the second immunization (Figure 4C). This observation indicates that a continuous decline of Treg/Tact ratio is mostly affected by the oligoclonal expansion of PG-specific CD25+CD4+ activated T cell, rather than a slight increase of Treg cells (Figure 4C).

## Discussion

The major goal of this study was to identify age-associated alterations in the functions and regulation of the immune system that may influence arthritis susceptibility in a genetically identical (inbred BALB/c) strain of mice. Increasing incidence of RA with age has been repeatedly shown in the human population [31]. Similarly, in the PGIA model of RA, we observed that adolescent young (1-month-old) mice are completely resistant to arthritis, but with age they become fully susceptible to the disease.

We found that T cell responses to the immunizing human PG (both proliferation and cytokine production) decreased with age, which, surprisingly, inversely correlated with arthritis onset and severity. In contrast to this, in a separate study on a limited number of mice, we found significantly lower autoreactive (mouse PG-specific) T cell responses in young than in old mice (F. Boldizsar and TT Glant, unpublished data). Collectively, these results suggest that, while in young mice an effective regulation of the immune response to PG still exists in older mice these mechanisms are partially lost. This "physiological" loss of control may lead to sustained activation of autoreactive T cells and auto-antibody production, directing the immune system against self antigen and culminating in joint inflammation in genetically susceptible animals.

Suppression of autoreactivity by Tregs has been shown in other autoimmune disease models [32-36]. Therefore, a candidate cell population that could be responsible for the age-related decline of the control over T cell reactivity in PGIA is Tregs. Indeed, in arthritis-resistant young mice, upon repeated PG injections, there was a Treg expansion, while in older mice the balance of regulatory/activated CD4+ cells shifted in favor of activated cells. The expansion of Tregs became especially evident locally in the peritoneal cavity after the second i.p. PG injection in 1-month-old mice (Figure 4). This period is critical in the development of PGIA, since the previously differentiated, potentially autoreactive cells are reactivated and the autoimmune attack on the joints begins at this time [28]. We believe that local Treg preponderance in younger mice (Figures 4A–B) at this stage of PG-immunization is responsible for the effective control of immune response, thus preventing autoimmunity.

Because PGIA is associated with Th1 dominance [27,37], special attention was given to the Th1/Th2 cytokine balance. The IFNγ: IL-4 ratio shifted toward the Th1 direction in the older, arthritis-susceptible mice, while in the arthritis-resistant 1-month-old group these two signature cytokines were produced in approximately equal amounts. IFNγ has been shown to be a key cytokine in PGIA, whereas IL-4 and IL-10 ameliorate the disease [27,37]. The counterbalance of these cytokines in PGIA has been shown in KO mice. While IFNγ-deficient BALB/c mice developed less severe arthritis, IL-4^-/- ^mice in the same background aggravated the response to PG immunization: an earlier onset with more severe inflammation was detected when compared to age- and gender-matched wild-type mice [38,39]. Our present study indicates that, although the amounts of both cytokines declined in aging animals, aging seems to lead to Th1 "dominance" upon PG immunization. However, whether the effective amounts of these cytokines or their ratio are more critical, remains an open question.

Besides the alterations in the PG-induced immune responses, age-related physiological changes of the immune system (maturation followed by immunosenescence) could also explain the differences in PGIA susceptibility of the different age groups. Therefore, we also examined the age-related immune system of naive, non-immunized, BALB/c mice. A detailed cellular analysis showed that the proportion of T cells decreased with age in the blood, and this was accompanied by an increase of the proportion of conventional B cells in the spleen and PLF of naive mice. This decreased T cell ratio was most likely due to the involution of the thymus, resulting in decreased thymic T cell output, and narrowing of the T cell repertoire [8]. On the other hand, the memory T cell pool showed expansion, while the naive T cell pool showed contraction in aging animals [7]. Indeed, a shift toward the memory phenotype (CD44^high^) in CD4+ T cells could be detected in the blood. The expression of CD28 co-stimulatory molecule decreased with age on helper T cells in the PLF, but not on spleen and blood T cells. Accumulation of CD28^null ^CD4+ T cells is often associated with immunosenescence [17]. The absence of this key costimulatory molecule is believed to result in perturbed T cell signaling, which could contribute to autoimmunity [17]. In sum, we conclude that the aging processes affect both T and B cells, which observations are in line with previous reports [7,40]. Since PGIA is both T- and B-cell (autoantibody)-dependent [24,41], the age-related cellular and phenotypic changes could profoundly affect arthritis susceptibility.

APCs play a critical role in initiating and regulating the immune response. Dendritic cells and B cells (as APCs) support Th1 polarization, while macrophages support the differentiation of Th2 effector cells [42]. Therefore, we also analyzed the age-related changes in the composition of APC populations in different compartments of the immune system upon i.p. PG immunization. In the peritoneum, there was a decrease of APCs of myeloid origin, and an increase of B cells in the first 3–5 months of age. In younger mice, there were more myeloid APCs (DCs and macrophages), while after 5 months, B cells dominated at the site of antigen inoculation (peritoneum). This shift in the APC composition coincided with an increase in arthritis susceptibility (around 3–5 months of age). The abundance of B cells observed in aged mice, most likely, supports Th1 dominance, and thus facilitates the development of PGIA.

## Conclusion

Taken together as a whole, our data revealed that with age, (i) the PG-immunization-induced T cell response decreased, (ii) and although the amounts of cytokines declined, Th1/Th2 cytokine balance was skewed toward Th1 direction, and (iii) the (auto)antibody production increased. Furthermore, in old mice, (iv) the T/B cell ratio decreased, (v) but there was a clear shift towards memory T cells, (vi) associated with a significant loss of CD28+CD4+ cells. Other potentially important components of immunosenescence in BALB/c mice included (vii) disturbed Treg induction upon PG immunization, (viii) several-fold increase of PG-activated T cells, and (ix) altered myeloid/lymphoid-APC ratio at the site of antigen injection (PLF).

In conclusion, immunosenescence is a physiological process that can alter immunoregulatory functions in aging individuals. We have identified some key aspects of the aging immune system that could play a role in the age-related autoimmune predisposition in BALB/c mice, thus affect arthritis susceptibility and severity. Complex age-related changes in T cell-APC interactions, and reduced generation of Tregs may lead to impaired immune regulation and development of autoimmune disease such as PGIA, making this murine model even more relevant to RA.

## Methods

### Antigens, animals, immunization, and sample collection

Human articular cartilage was collected from patients who underwent knee joint replacement surgery. The collection of cartilage from consenting patients was approved by the Institutional Review Board of Rush University Medical Center (Chicago). Cartilage PG (aggrecan) was extracted and depleted of glycosaminoglycan side chains as described [25]. Mouse cartilage PG was prepared by CsCl gradient centrifugation as described [23,43].

All animal procedures were conducted under a protocol approved by the Institutional Animal Care and Use Committee of Rush University Medical Center. BALB/c mice from Charles River Laboratories (Kingston Colony) were bred in-house under specific pathogen-free conditions. Siblings of 1 to 11 months of age were immunized intraperitoneally (i.p.) with an emulsion of cartilage PG (100 μg protein) and 2 mg dimethyldioctadecyl-ammonium bromide (DDA) adjuvant on days 0, 21 and 42 [25]. Five animals from each age group were sacrificed prior to immunization (naive, non-immunized mice), or 10–15 days after the first, second and third immunizations. The remaining animals were sacrificed 87 days after the first PG/DDA injection (Table 1). Heparinized blood samples, cells of peritoneal lavage fluid (PLF) and spleen cells were collected at the time of sacrifice.

### Clinical assessment of arthritis

PG-immunized mice were examined 3 times a week for clinical symptoms of arthritis after the second immunization. The time of onset and incidence of arthritis were recorded, and severity was scored based upon swelling and redness of each paw ranging from 0 to 4, yielding a maximum severity score of 16 per mouse [20,25,41].

### Measurement of antigen-specific antibodies and T cell responses

Serum samples and spleen cells were collected from naive (non-immunized), and all immunized mice 87 days after the first PG injection. PG-specific antibodies were measured by enzyme-linked immunosorbent assay (ELISA) as described [25,29]. Sera were diluted at the range of 1:100–1:102,400, and PG-specific antibodies were detected with peroxidase-conjugated rabbit anti-mouse IgG1 and IgG2a, respectively (Zymed Laboratories, San Francisco, California, USA).

Antigen-specific T cell responses were measured in quadruplicate samples of spleen cells (3 × 10^5 ^cells/well) cultured in the presence of 50 μg human PG protein/ml or, in an additional experiment, 25 μg mouse PG protein/ml. IL-2 was measured on day 2 in 100 μl supernatant of PG-stimulated spleen cell cultures using a CTLL-2 bioassay [44]. T cell proliferation was assessed using [^3^H]thymidine incorporation into spleen cells on day 5 [29,45]. Spontaneous and antigen-specific production of IL-4, IL-6, IL-17, TNFα, and IFNγ were measured in cell culture supernatants (1.8 × 10^6 ^cells/well) on day 5 using capture ELISA (BD Biosciences, San Jose, California, USA or R&D Systems, Minneapolis, Minnesota, USA) and the results were expressed as pg amounts of cytokine secreted by 1 × 10^6 ^cells [45].

### Chemicals and monoclonal antibodies (mAbs)

All chemicals, unless indicated otherwise, were purchased from Sigma Chemical Co. (St. Louis, Missouri, USA) or Fischer Scientific (Chicago, Illinois, USA). Mouse recombinant cytokines and ELISA kits were purchased from R&D Systems or BD Biosciences. Phosphate buffered saline (PBS) was used for washing and storing cells until use. Cell surface labeling with mAbs was carried out in flow buffer [PBS containing 0.1% NaN_3_, 0.1% bovine serum albumin (BSA)]. FoxP3 labeling was performed in permeabilization buffer (eBiosciences, San Diego, CA) after fixation of cells in fixation/permeabilization buffer (eBiosciences).

The following mAbs purchased from BD Biosciences were used: PerCP-Cy5.5-conjugated rat anti-mouse CD4 (clone RM4-5), PE-Cy7-conjugated rat anti-mouse B220 (clone RA3-6B2), APC-conjugated rat anti-mouse CD25 (clone PC61), APC-Cy7-conjugated Armenian hamster anti-mouse CD3 (clone 145-2C11), FITC-conjugated rat anti-mouse IgD (clone 11-26c.2a), PE-conjugated rat anti-mouse CD43 (clone S7), PerCP-Cy5.5-conjugated rat anti-mouse IgM (clone R6-60.2), PE-Cy7-conjugated rat anti-mouse CD19 (clone 1D3), APC-conjugated rat anti-mouse CD5 (clone 53-7.3), biotin-conjugated rat anti-mouse CD23 (clone B3B4), Alexa Fluor 488-conjugated rat anti-mouse CD44 (clone IM7), and PE-conjugated rat anti-mouse CD62L (clone MEL-14). The PE-conjugated rat anti-mouse FoxP3 (clone FJK-16s), APC-conjugated hamster anti-mouse CD28 (clone 37.51), APC-conjugated rat anti-mouse F4/80 (clone BM8). PE-Cy7-conjugated Armenian hamster anti-mouse CD11c (clone N418) was purchased from eBiosciences.

### Flow cytometry

We used a multicolor labeling technique for the simultaneous detection of cell surface and intracellular molecules on PLF, spleen, and peripheral blood leukocytes. Briefly, 1 × 10^6 ^cells were seeded in 96-well U-bottom assay plates (BD Falcon), Fc receptors blocked at 4°C in the dark for 15 min, and then incubated with mAb cocktails in 100 μl flow cytometry staining buffer at 4°C in the dark for 30 min. Cells were washed twice, and finally resuspended in 200 μl of 0.1% buffered formaldehyde. If biotinylated mAbs were used, after washing, fluorochrome-labeled streptavidin was added to the samples and incubated at 4°C in the dark for another 30 min. FoxP3 was stained after fixation and permeabilization of cells. Isotype-matched control Abs for each specific mAb was used for background staining in each experiment.

Samples were measured and analyzed in a FACS Canto II flow cytometer (Becton Dickinson, San Jose, CA) using a high-throughput-microplate module platform and DIVA software. Initial gating was performed on lymphoid cells based on forward/side scattering (FSC/SSC) properties. Cell surface marker-defined populations were as follows: CD3+: total T cells; CD3+ CD4+: CD4+ T cells; CD3+ CD8+: CD8+ T cells; CD4+ CD25+ FoxP3+: Tregs; B220+: total B cells; IgD^low ^IgM^high ^CD19+ CD23- CD5+/-: B1 cells (B1a/b); IgD^high ^IgM^low ^CD19+ CD23-: B2 cells [46]; F4/80+: macrophages; and CD11c+: dendritic cells. We collected data of 10,000 cells from the lymphoid gate in each sample, unless otherwise stated. We used fluorescent histogram-, dot- and contour plots both for comparing mean fluorescence intensities of different samples and calculating the ratio of positively stained cells.

### Statistical analysis

Descriptive statistics were used to determine group means and standard error of the mean (mean ± SEM). The differences between multiple groups were tested for statistical significance using ANOVA. Two-tailed Pearson-correlation analysis was used to find correlations between data sets. A p < 0.05 value was considered to be statistically significant.

## Abbreviations

APC: antigen presenting cells; DC: dendritic cells; DDA: dimethyldioctadecyl-ammonium bromide; mAb: monoclonal antibody; PG: cartilage proteoglycan aggrecan; PGIA: PG-induced arthritis; PLF: peritoneal lavage fluid; RA: rheumatoid arthritis; Treg: regulatory (CD25^high ^FoxP3+) T cells; Tact: activated (CD25^high^) T cells.

## Competing interests

The authors declare that they have no competing interests.

## Authors' contributions

OT performed the research, controlled phenotypic analysis and performed most of the assays (antibody measurements, T-cell responses and cytokine Elisa's. She was actively involved in preparation of figures and manuscript. FB performed all flow cytometry measurements, analyzed data and put together the first (draft) version of the manuscript. KM and PN actively involved in all aspect of experimental design and finalized the content of the manuscript, while TTG conceived the study, participated in its design, coordinated animal breeding and laboratory experiments, and finalized and submitted the manuscript. All authors read and approved the final manuscript.
